# The ability of four frailty screening instruments to predict mortality, hospitalization and dependency in (instrumental) activities of daily living

**DOI:** 10.1007/s10433-019-00502-4

**Published:** 2019-02-19

**Authors:** Linda P. M. Op het Veld, Anna J. H. M. Beurskens, Henrica C. W. de Vet, Sander M. J. van Kuijk, KlaasJan Hajema, Gertrudis I. J. M. Kempen, Erik van Rossum

**Affiliations:** 1 Centre of Research Autonomy and Participation for Persons with a Chronic Illness, Faculty of Health, Zuyd University of Applied Sciences, P.O. Box 550, 6400 AN Heerlen, The Netherlands; 2 CAPHRI, Care and Public Health Research Institute, Department of Health Services Research, Maastricht University, P.O. Box 616, 6200 MD Maastricht, The Netherlands; 3 CAPHRI, Care and Public Health Research Institute, Department of Family Practice, Maastricht University, P.O. Box 616, 6200 MD Maastricht, The Netherlands; 4 Department of Epidemiology and Biostatistics, Amsterdam Public Health Research Institute, Amsterdam University Medical Centers, location VU University, De Boelelaan 1089A, 1081 HV Amsterdam, The Netherlands; 5 Department of Clinical Epidemiology and Medical Technology Assessment, Maastricht University Medical Centre, P.O. Box 5800, 6202 AZ Maastricht, The Netherlands; 6 Community Health Service South Limburg, Academic Collaborative Centres Public Health (ACC), P.O. Box 33, 6400 AA Heerlen, The Netherlands

**Keywords:** Frail older people, Frailty, Predictive value of tests, Screening, Sensitivity, Specificity

## Abstract

The aim of this study was to assess the predictive ability of the frailty phenotype (FP), Groningen Frailty Indicator (GFI), Tilburg Frailty Indicator (TFI) and frailty index (FI) for the outcomes mortality, hospitalization and increase in dependency in (instrumental) activities of daily living ((I)ADL) among older persons. This prospective cohort study with 2-year follow-up included 2420 Dutch community-dwelling older people (65+, mean age 76.3 ± 6.6 years, 39.5% male) who were pre-frail or frail according to the FP. Mortality data were obtained from Statistics Netherlands. All other data were self-reported. Area under the receiver operating characteristic curves (AUC) was calculated for each frailty instrument and outcome measure. The prevalence of frailty, sensitivity and specificity were calculated using cutoff values proposed by the developers and cutoff values one above and one below the proposed ones (0.05 for FI). All frailty instruments poorly predicted mortality, hospitalization and (I)ADL dependency (AUCs between 0.62–0.65, 0.59–0.63 and 0.60–0.64, respectively). Prevalence estimates of frailty in this population varied between 22.2% (FP) and 64.8% (TFI). The FP and FI showed higher levels of specificity, whereas sensitivity was higher for the GFI and TFI. Using a different cutoff point considerably changed the prevalence, sensitivity and specificity. In conclusion, the predictive ability of the FP, GFI, TFI and FI was poor for all outcomes in a population of pre-frail and frail community-dwelling older people. The FP and the FI showed higher values of specificity, whereas sensitivity was higher for the GFI and TFI.

## Introduction

Over the past decades, many instruments have been developed to identify frail older people (Pialoux et al. 2012). Since consensus on a frailty definition is still lacking, these instruments are based on different concepts. For example, Fried and colleagues proposed an instrument based on (five) solely physical measures, the Frailty Phenotype (FP) (Fried et al. 2001). Others prefer a broader concept and also include other, predefined domains, such as social or psychological domains, in their frailty instrument. An example of the latter is the Tilburg Frailty Indicator (TFI), developed by Gobbens et al. (2010). Rockwood and Mitnitski (2007) also proposed a multi-domain concept with their Frailty Index (FI). In contrast to the frailty measures with predefined domains, the FI is characterized by a non-fixed set of items of so-called deficits. The common factor of all of these instruments, irrespective of the frailty definition used, is that when a person is classified as frail, there is an increased risk of adverse outcomes, such as mortality, disability, institutionalization and hospitalization (Sternberg et al. 2011).

A fair amount of research has been conducted on the predictive validity of frailty instruments (Apostolo et al. 2017; Pijpers et al. 2012). Nevertheless, much variation exists, for instance in study setting (community-dwelling, assisted living, hospitalized) (Coelho et al. 2015; Hogan et al. 2012; Warnier et al. 2017), outcomes (e.g., death, disability, institutionalization, hospitalization, falls) (Sternberg et al. 2011), follow-up period (ranging from a few weeks to several years) (Daniels et al. 2012; Fried et al. 2001), ethnicities (e.g., African-American, Mexican-American) (Graham et al. 2009; Malmstrom et al. 2014) and gender (males, females or both) (Papachristou et al. 2017; Sternberg et al. 2011). If only one instrument is included in a study, the aforementioned variation makes it difficult to compare the predictive accuracy of different frailty instruments. Several studies have examined two or more instruments in one population. For example, Theou et al. (2013) compared eight different frailty instruments with regard to their ability to predict all-cause mortality.

Two instruments that are frequently used worldwide are the FP and the FI. In the Netherlands and other European countries, the multi-dimensional Groningen Frailty Indicator (GFI) and TFI with fixed sets of questions are often used in particular. However, the predictive ability of these instruments has not been thoroughly tested before in one population with the same, multiple outcomes and within the same timeframe (Theou et al. 2013).

The aim of this study was to investigate and compare the predictive ability of the four aforementioned frailty instruments for the outcomes mortality, hospitalization and increase in (I)ADL dependency, in a large sample of community-dwelling older people in the Netherlands.

## Methods

A prospective cohort study with a 2-year follow-up period was conducted (Polit and Beck 2016). The study was approved by the medical ethical committee of Zuyderland and Zuyd University of Applied Sciences (METC Z, 12-N-129).

### Selection of participants

The Dutch Community Health Services sent out an extensive general health questionnaire to 56,000 people aged 55 years and over in the Province of Limburg, a southern region of the Netherlands in 2012. Of the respondents to this questionnaire, pre-frail or frail individuals (according to Fried’s frailty criteria) who were at least 65 years old were asked to participate in our study. The selection of this cohort is described in detail elsewhere (Op Het Veld et al. 2017). In total, 2420 persons gave their informed consent and participated in our study.

### Data collection

Demographic data (i.e., gender, age) were collected at baseline, along with four frailty measures. The occurrence of three different outcome measures was determined at 2-year follow-up.

### Frailty measures

Four frailty instruments were investigated in this study. The FP, GFI and TFI all have been validated among community-dwelling older people (Fried et al. 2001; Gobbens et al. 2010; Peters et al. 2012). The FI that we developed has not been validated yet; however, FI’s in general have shown to be a valid frailty instrument among community-dwelling older people (Drubbel et al. 2013; Mitnitski et al. 2001).

### Frailty Phenotype (FP)

Fried and colleagues described five physical criteria (weight loss, exhaustion, physical activity, walk time and handgrip strength) for the identification of frail older people (Fried et al. 2001). Weight loss, exhaustion and physical activity are self-report questions, whereas walk time and handgrip strength are originally performance-based measures. A partially modified version of these criteria was used in this study. In short, physical activity was measured with a slightly adjusted version of the Short Questionnaire to Assess Health-enhancing physical activity (SQUASH) (Wendel-Vos et al. 2003). The performance-based measures were unfeasible in this large-scale study, and therefore substituted by self-report questions. More details of the self-report measurement of these criteria are described elsewhere (Op het Veld et al. 2015). Theoretical scores range from 0 to 5, classifying individuals as non-frail (score 0), pre-frail (score 1–2) or frail (score 3–5). Only pre-frail and frail persons were included in this study (see above).

### Groningen Frailty Indicator (GFI)

The GFI, developed by Steverink et al. (2001), is a frailty screening instrument consisting of fifteen self-report questions focusing on multiple domains of functioning: physical (9 items), cognitive (1 item), social (3 items) and psychological (2 items). Theoretical scores range from 0 (no frailty) to 15 where persons with a score ≥ 4 are considered frail (Schuurmans et al. 2004).

### Tilburg Frailty Indicator (TFI)

The TFI was developed by Gobbens et al. (2010). It consists of two parts: Part A comprises determinants of frailty, such as socio-demographic data and data about chronic diseases, while Part B, which determines the level of frailty, is used in the present study and comprises a total of 15 questions on multiple domains: physical (8 items), psychological (4 items) and social (3 items). Theoretical scores derived from Part B range from 0 (no frailty) to 15. A person is considered frail with a score of ≥ 5 (Gobbens et al. 2010).

### Frailty Index (FI)

The Frailty Index is characterized by a non-fixed set of ‘deficits’ (Rockwood and Mitnitski 2007). To create a frailty index, we used the guidelines described by Searle et al. (2008). Sixty-one potential items were selected from the extensive questionnaire sent by the Dutch Community Health Services. All items were dichotomized, where a score of ‘0’ indicated the absence and a score of ‘1’ the presence of the deficit. Next, all items with a prevalence of less than five percent were excluded, as proposed in a previous study (Drubbel et al. 2013). The final Frailty Index consisted of 53 items, covering several topics, such as (chronic) diseases, loneliness, physical limitations and psychological distress. A cutoff value of 0.25 (which is equal to a positive score on 25% of the total number of items), as proposed by the original authors, was used to distinguish between frail and non-frail individuals (Rockwood et al. 2007).

### Outcome measures

Outcome measures were mortality, hospitalization and an increase in (I)ADL dependency. Statistics Netherlands provided mortality data (deceased yes/no) at the 2-year follow-up.

Self-report follow-up questionnaires were used to gather information about hospitalization (every 6 months) and (I)ADL dependency (at 2-year follow-up). For hospitalization, every 6 months the study participants were asked whether they had been admitted to a hospital in the previous 6 months. Participants were divided into two groups: those who reported a hospital admission at least once and those who reported no hospital admission at any of the time points during the 2-year observation period. To determine the level of (I)ADL dependency, the Groningen Activity Restriction Scale (GARS) (Kempen et al. 1996) was measured at baseline and after 2 years. The GARS comprises 18 questions about the degree to which someone is able to perform ADL and IADL activities independently. The four response options are: ‘Yes, I can do it fully independently without any difficulty’, ‘Yes, I can do it fully independently but with some difficulty’, ‘Yes, I can do it fully independently but with great difficulty’, ‘No, I cannot do it fully independently, I can only do it with someone’s help’. Results were first dichotomized into being independent (the first three options) or dependent (the fourth option) regarding the performance of activities, as described in the GARS manual (Kempen et al. 2012). We chose this way of analyzing because losing one’s independency is particularly critical and has a higher impact on people’s lives than having difficulties (without dependency) in performing (I)ADL. Then, changes over time per item were analyzed. When someone changed from independent to dependent more often than from dependent to independent, a positive score was assigned to the outcome (I)ADL dependency. This means that someone experienced a higher level of dependency in performing (I)ADL activities over the 2-year observation period.

### Statistical analysis

Descriptive statistics were computed to provide an overview of the study population.

As proposed in previous research, one missing value of the FP was allowed when a person had a valid score of 0–2 and two missing values were allowed if the FP score was ≥ 3 (Op het Veld et al. 2015). As suggested by Metzelthin et al. (2010), missing items of the GFI and TFI were imputed by means of case mean substitution, if less than 25% of all items were missing. Case mean substitution was applied for the GARS if less than 50% of the total number of items were missing (Kempen et al. 1996). Missing values for each item of the FI were imputed using the non-missing population mean of that item, as proposed by the developers (Song et al. 2010).

Per screening tool, we created receiver operating characteristic (ROC) curves based on the continuous scores of the instrument and calculated the area under the ROC curve (AUC) per outcome measure to assess the predictive validity. We consider an AUC of 0.90–1 being excellent, 0.80–0.90 being good, 0.70–0.80 being fair, 0.60–0.70 being poor and 0.50–0.60 non-informative. Next, the prevalence of frailty, sensitivity and specificity were calculated for each frailty instrument and for each outcome measure, using the cutoff values as proposed by the developers and for the cutoff values one above and one below the proposed values (0.05 for the FI). All statistical analyses were performed using IBM SPSS Statistics for Windows version 22.

## Results

A total of 2420 persons (mean age 76.3 ± 6.6 years, 39.5% male), who were pre-frail or frail according to Fried’s frailty score, participated in this study. Characteristics of the study population are described in Table 1.

**Table 1:** Characteristics of the study population at baseline (*n* = 2420)

Variable	Value	Observed range
Gender (male, %)	957 (39.5%)	
Age (mean ± SD)	76.3 ± 6.6	65–97
FP (*n*, %)		
1	1317 (54.4%)	
2	566 (23.4%)	
3	358 (14.8%)	
4	153 (6.3%)	
5	26 (1.1%)	
GFI (**0**–15)^a^ (mean ± SD)	4.58 ± 2.97	0–14
TFI (**0**–15)^a^ (mean ± SD)	5.97 ± 3.31	0–15
FI (**0**–1)^a^ (mean ± SD)	0.20 ± 0.12	0–0.76

*FP* Frailty Phenotype, *GFI* Groningen Frailty Indicator, *TFI* Tilburg Frailty Indicator, *FI* Frailty Index, *SD* standard deviation

^a^Theoretical range, preferable score is bolded

After 2 years, 182 (7.5%) participants had died, about one third (*n* = 836) had been admitted to a hospital at least once, and 668 participants had experienced a higher level of (I)ADL dependency.

**Fig. 1:**
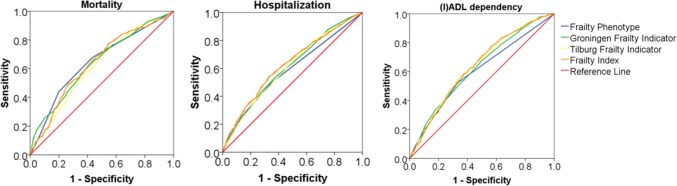
Receiver operating characteristic (ROC) curves for all frailty instruments and per outcome measure

First, to assess the predictive ability of the frailty instruments, ROC curves were plotted (Fig. 1) and the areas under these curves were calculated (Table 2) for each instrument and each outcome measure. Per outcome measure, the AUCs of all instruments were fairly similar; the AUCs of all four frailty instruments for the prediction of mortality, hospitalization and (I)ADL dependency were poor (AUCs between 0.62–0.65, 0.59–0.63 and 0.60–0.64, respectively).

**Table 2:** Area under the ROC curve per frailty instrument and for each outcome measure

Frailty instrument	Mortality^a^	Hospitalization^a^	(I)ADL dependency^a^
FP	0.65 (0.61–0.69)	0.59 (0.56–0.61)	0.60 (0.57–0.63)
GFI	0.64 (0.60–0.68)	0.61 (0.58–0.64)	0.63 (0.60–0.65)
TFI	0.62 (0.58–0.66)	0.61 (0.58–0.63)	0.64 (0.61–0.66)
FI	0.64 (0.60–0.68)	0.63 (0.60–0.65)	0.64 (0.61–0.66)

(*I*) *ADL* (instrumental) activities of daily living, *FP* Frailty Phenotype, *GFI* Groningen Frailty Indicator, *TFI* Tilburg Frailty Indicator, *FI* Frailty Index

^a^Area under the curve (95% confidence interval)

Next, based on the cutoff value proposed by the developers, the prevalence of frail participants was calculated for each frailty instrument, as well as the associated sensitivity and specificity for each outcome measure (Table 3). The prevalence of frail participants in this population (pre-frail and frail individuals according to the FP) varied from 22.2% (FP) to 64.8% (TFI). Regarding the proposed cutoffs, the FP and the FI showed higher levels of specificity compared to sensitivity for all outcome measures. Specificity was fairly similar for both instruments (FP range 79.6–86.2%, FI 71.4–79.6%). In contrast, the GFI and TFI had higher levels of sensitivity compared to specificity for all outcome measures. The sensitivity of these two frailty instruments varied more between outcome measures than specificity for the FP and FI. Sensitivity of the GFI and TFI was 76.2% and 80.6%, respectively, for mortality, lower for (I)ADL dependency (GFI 66.0%, TFI 72.7%) and lowest for hospitalization (GFI 63.9%, TFI 70.5%).

**Table 3:** Prevalence, sensitivity and specificity for different cutoffs of FP, GFI, TFI and FI for mortality, hospitalization and (I)ADL dependency among pre-frail and frail older people

Frailty instrument	Cutoff	Frail (*n*)	Frail (%)	Mortality		Hospitalization		(I)ADL dependency	
				Sens^a^	Spec^b^	Sens^a^	Spec^b^	Sens^a^	Spec^b^
FP	≥ 2	1103	45.6	68.1	56.3	50.2	64.0	52.8	66.0
	**≥** **3**	**537**	**22.2**	**44.5**	**79.6**	**25.6**	**86.2**	**24.7**	**86.2**
	≥ 4	179	7.4	13.7	93.1	8.7	96.4	8.5	96.2
GFI	≥ 3	1697	70.7	82.3	30.3	75.2	37.1	77.5	37.9
	**≥** **4**	**1424**	**59.3**	**76.2**	**42.1**	**63.9**	**50.3**	**66.0**	**51.1**
	≥ 5	1150	47.9	66.3	53.6	53.5	62.9	53.8	63.1
TFI	≥ 4	1743	73.5	86.1	27.5	79.6	34.7	82.2	36.3
	**≥** **5**	**1536**	**64.8**	**80.6**	**36.5**	**70.5**	**44.1**	**72.7**	**45.7**
	≥ 6	1280	54.0	72.2	47.5	59.7	54.8	62.8	57.6
FI	≥ 0.20	1079	44.6	64.8	57.0	52.3	67.4	51.8	67.4
	**≥** **0.25**	**730**	**30.2**	**49.5**	**71.4**	**35.9**	**79.6**	**34.3**	**79.2**
	≥ 0.30	484	20.0	32.6	81.0	24.8	87.7	23.4	87.1

Cutoff values as proposed by the original authors are highlighted in bold

*(I)ADL* (instrumental) activities of daily living, *FP* Frailty Phenotype, *GFI* Groningen Frailty Indicator, *TFI* Tilburg Frailty Indicator, *FI* Frailty Index

^a^Sensitivity (%)

^b^Specificity (%)

The same analyses were conducted with the cutoff value one point above or below the proposed cutoff value (0.05 for the FI) (Table 3). Using a lower or higher cutoff value than that proposed by the original authors considerably changes the sensitivity and specificity of each frailty instrument.

## Discussion

The aim of this study was to investigate the ability of four frailty instruments to predict mortality, hospitalization and an increase in (I)ADL dependency over a 2-year time period among pre-frail and frail community-dwelling older people. The predictive ability of all included frailty instruments was poor for the outcomes mortality, hospitalization and (I)ADL dependency (AUCs between 0.59 and 0.65). The Frailty Phenotype and the Frailty Index showed higher values for specificity, while the Groningen Frailty Indicator and Tilburg Frailty Indicator had higher values for sensitivity. This indicates that the GFI and TFI are more able to correctly identify frail people as frail, whereas the FP and FI seem to be better at identifying non-frail people as such.

The AUCs in our study are low, and whether they can be considered clinically meaningful can be argued. Nevertheless, despite the fact that we used a study population with only pre-frail and frail individuals, our results are fairly in line with previous research. For example, Daniels et al. (2012) investigated the GFI and TFI in a 1-year follow-up study and found AUCs of 0.64 and 0.64, respectively, for mortality, 0.54 and 0.60 for hospitalization and 0.67 and 0.66 for the development of disabilities. Also Widagdo et al. (2015) found comparable values for the FP in predicting mortality (AUC 0.57) and hospitalization (AUC 0.52) and for the FI in predicting mortality (AUC 0.60) and hospitalization (0.56). Theou et al. (2013) reported higher values of all four frailty instruments for the prediction of mortality at 2-year follow-up (AUCs between 0.72 and 0.77). Their population was younger (50+, mean age 65.3 ± 10.5 years) and also included non-frail persons. The FP, GFI and TFI that they used were modified versions with data derived from one questionnaire. However, it is not known to what extent this could explain the differences in AUC. In our study, all instruments were least able to predict hospitalization, which is in line with other studies (Daniels et al. 2012; Widagdo et al. 2015). Admission to a hospital depends on more factors than only frailty, such as availability of (informal) care, distance to healthcare facilities et cetera.

Although the AUC per outcome measure was fairly comparable between instruments, differences were found in the values of sensitivity and specificity. The GFI and TFI had higher values of sensitivity, which indicates that they are more able to correctly classify frail participants as being frail. These results are not fully in line with the study of Daniels et al. (2012). They found values of sensitivity and specificity that were closer to each other (i.e., no high sensitivity with a low specificity or vice versa) than in our study. Also, for hospitalization, a higher specificity was reported compared to sensitivity for both the GFI and TFI, as well as a higher specificity for the TFI with regard to the development of disabilities. Gobbens et al. (2012) also investigated the predictive ability of the TFI for the outcome hospitalization over a 2-year period. They found higher specificity values, whereas we found higher values for sensitivity. Contradictory to the GFI and TFI in our study, the FP and FI had higher values of specificity, which indicates that they are more able to correctly classify non-frail participants as such. Similarly, Widagdo et al. (2015) found higher levels of specificity for the FP and FI in the prediction of mortality and hospitalization. In general, the results that we presented in Table 3 show that using different conceptualizations of frailty by the four screening instruments and the associated outcome measures, results in a large variation regarding prevalence rates and predictive values, which has also been demonstrated by previous research on frailty (Collard et al. 2012; Daniels et al. 2012).

Considering the fact that none of the instruments in our study had both high sensitivity and specificity, nor when the cutoff values were increased or decreased, choosing an instrument for use in research or daily practice depends on the goals that one aims to achieve. For example, if one wants to include frail persons into an intervention program, a highly specific test should be used. False-positive rates will be low, however, some frail persons will be missed (false-negative). A highly sensitive test has few false-negative results and should be chosen when one does not want to miss any frail person, but such an instrument also includes more non-frail persons (false-positive). When even higher values of either sensitivity or specificity are required, the used cutoff point of a specific instrument can be changed. Another point of consideration when choosing an instrument is the time that is needed for filling out the questionnaire. Most questionnaires are relatively short, however, the FI comprises many items and might therefore seem less suitable. Nevertheless, often a FI can be (automatically) calculated using readily available information from patients records from, for example, general practices or hospitals. Then, the FI can be easily used as a screening instrument.

The strength of the present study is that it was conducted in a large, well-defined sample of community-dwelling older people. Moreover, four instruments were analyzed using the same population with three outcome measures and within the same timeframe of 2 years. It should be noted that the FP was partially modified, which might have influenced the results. Only pre-frail and frail individuals were included in this study. Our target population was a population at risk. In daily practice, frailty instruments are most often applied by healthcare professionals. People that already make use of healthcare services are more likely to be (pre-)frail (Op het Veld et al. 2015) and therefore at risk. Hence, the inclusion of pre-frail and frail persons in our study makes our population more reflective of the persons for whom frailty measures are useful, than for a large sample of the general population. Consequently, prevalence rates in our study might differ from the ones found in studies that included samples from a general older population. Also, sensitivity, specificity and AUC might be somewhat smaller due to the choice of a more challenging, yet we think more adequate, population. The AUC of the GFI for the outcome (I)ADL dependency was, at least to some extent, overestimated because four items of the GFI resembled items included in the GARS, the latter which was used as the (I)ADL dependency measure. The same holds for the results of the FI for the outcome (I)ADL dependency, since six out of the 53 items were similar to GARS items. Another factor that could possibly have affected the results of the study is that, except for mortality, all data are based on self-report questionnaires. We cannot rule out recall bias (e.g., with respect to hospitalization in the last 6 months) or bias due to cognitive limitations.

The four studied frailty instruments only poorly predicted mortality, hospitalization and an increase in (I)ADL dependency. As more people become frail and suffer from adverse outcomes, the need for intervention programs is increasing. In order to be able to include or exclude the right target group in these intervention programs, it is important to screen effectively. Previous studies suggested the combined use of frailty measures, for instance, a combination of the Frailty Phenotype and the Frailty Index (Cesari et al. 2014; Dent et al. 2016). Our study shows that these instruments both have higher specificity rates. It might be suggested that the combination of an instrument with a high specificity (FI or FP) and one with a high sensitivity (GFI or TFI) would result in a better identification of frail older people and a better prediction of adverse outcomes. Future research could be aimed at investigating the use of several combinations of existing frailty instruments. Another option is to combine individual items of (two or more) existing questionnaires and use this as a starting point for the creation of a new frailty instrument, with preference for items with the highest predictive ability for serious outcomes. Also a different use of instruments, such as the frailty subtypes derived from the FP that were described by Liu et al. (2017), might increase the predictive ability.

In conclusion, the predictive ability of the FP, GFI, TFI and FI was poor for the outcomes mortality, hospitalization and increase in (I)ADL dependency in a population of pre-frail and frail community-dwelling older people. The FP and the FI showed higher values of specificity, whereas the GFI and TFI had higher values of sensitivity.
